# Association of Tag SNPs and Rare CNVs of the MIR155HG/miR-155 Gene with Epilepsy in the Chinese Han Population

**DOI:** 10.1155/2015/837213

**Published:** 2015-09-06

**Authors:** Hua Tao, Lili Cui, You Li, Xu Zhou, Guoda Ma, Lifen Yao, Jiawu Fu, Wen Li, Yujie Cai, Haihong Zhou, Wangtao Zhong, Shuyan Zhang, Zhien Xu, Keshen Li, Bin Zhao

**Affiliations:** ^1^Department of Neurology, Affiliated Hospital of Guangdong Medical College, Zhanjiang, Guangdong 524001, China; ^2^Institute of Neurology, Guangdong Medical College, Zhanjiang, Guangdong 524001, China; ^3^Clinical Research Center, Guangdong Medical College, Zhanjiang, Guangdong 524001, China; ^4^Department of Neurology, The First Affiliated Hospital of Harbin Medical University, Harbin, Heilongjiang 150001, China; ^5^Department of Neurology, The Fourth Affiliated Hospital of Harbin Medical University, Harbin, Heilongjiang 150001, China

## Abstract

*Background*. miR-155 likely acts as an important modulator in the inflammatory mechanism of epilepsy, and this study investigated its association with epilepsy from the perspective of molecular genetics.* Methods*. This study enrolled 249 epileptic patients and 289 healthy individuals of the Chinese Han population; 4 tag single-nucleotide polymorphisms (SNPs: rs969885, rs12483428, rs987195, and rs4817027) of the MIR155HG/miR-155 gene were selected, and their association with epilepsy was investigated. Additionally, this study determined the copy numbers of the MIR155HG/miR-155 gene.* Results*. The TCA haplotype (rs12483428-rs987195-rs4817027) and the AA genotype at rs4817027 conferred higher vulnerability to epilepsy in males. Stratification by age of onset revealed that the CC haplotype (rs969885-rs987195) was a genetic susceptibility factor for early-onset epilepsy. Further stratification by drug-resistant status indicated the CC haplotype (rs969885-rs987195) and the AA genotype at rs4817027 were genetic susceptibility factors for drug-resistant epilepsy (DRE) but the CG haplotype (rs987195-rs969885) was a genetically protective factor against DRE. Besides, 3 epileptic patients with copy number variants of the MIR155HG/miR-155 gene were observed.* Conclusions*. This study first demonstrates the association of MIR155HG/miR-155 tag SNPs with epilepsy and shows that rare CNVs were found exclusively in epileptic patients, clarifying the genetic role of miR-155 in epilepsy.

## 1. Introduction

Epilepsy is characterized by recurrent and unpredictable seizures and affects 65 million people worldwide. Although the new generation of antiepileptic drugs (AEDs) is widely applied in clinical settings, approximately 30% of these patients are still refractory [1]. Notably, inflammation has become a crucial mechanism of epilepsy in recent years [2–5], uncovering in depth the mechanism that might contribute to the prevention of epileptic processes and the development of new approaches for resistance.

MicroRNAs (miRNAs), a key subset of noncoding RNAs, exhibit biological functions by inhibiting the expression of their targets at the posttranscriptional level. Previous studies have shown that miRNAs extensively participate in the regulation of inflammatory diseases, as shown, for example, for miR-146a in Aronica's paper and for miR-133*α* in Law's paper [6–8]. Additionally, miR-155, one of the most intensively studied miRNAs, plays an inflammatory role in peripheral diseases, such as rheumatoid arthritis and ulcerative colitis [9, 10]. Microglia is the first line of the inflammatory response in the central nervous system, and the expression of miR-155 increases after microglia is exposed to the inflammatory stimulus lipopolysaccharide. This effect is accompanied by a lower level of suppressor of cytokine signaling 1 (SOCS1), a predicted target of miR-155 and a key inhibitor of the inflammatory process via the JAK/STAT (Janus kinase/signal transducer and activator of transcription) pathway [11]. After knockdown of miR-155 using anti-miRNA oligonucleotides, the expression of SOCS1 and its downstream inflammatory cytokines increased significantly [11]. Another study further demonstrated that miR-155 was upregulated in the hippocampus in a rat epileptic model and in patients with mesial temporal lobe epilepsy (MTLE) [12]. Hence, miR-155 likely acts as an important modulator in the inflammatory mechanism of epilepsy.

Single-nucleotide polymorphisms (SNPs), the most common type of genetic variations, influence susceptibility to disease by altering the expression of related genes. Because miR-155 is a transcription product of its host gene (MIR155HG), its expression could be affected by genetic variations of the MIR155HG gene as well as of the miR-155 gene. To date, two studies have reported the association of MIR155HG/miR-155 SNPs with multiple sclerosis and atopic eczema, and only the GTT haplotype (rs2829803-rs2282471-rs2829806) has been successfully identified as a genetic susceptibility factor for multiple sclerosis [13, 14]. However, the relation of MIR155HG/miR-155 SNPs with epilepsy is still undetermined. Another important type of genetic variations is copy number variations (CNVs), which mainly include duplication and deletion of DNA fragments [15]. Interestingly, several studies have found an association between a hot spot (21q21) of duplication and susceptibility to epilepsy [16, 17], and miR-155 is encoded in this region; thus the copy numbers of the MIR155HG/miR-155 gene might increase in epileptic patients. In this respect, it is reasonable to speculate that upregulated expression of miR-155 in epileptic patients might result from dosage effects of its duplication in 21q21, which then leads to increased susceptibility to epileptic seizures.

In the present study, we aimed to explore the association between miR-155 and epilepsy from the perspective of molecular genetics, including SNPs and CNVs, to clarify the genetic role of miR-155 in the generation of epilepsy.

## 2. Materials and Methods

### 2.1. Ethics Statement

This study was approved by the Ethics Committees of the Affiliated Hospital of Guangdong Medical College and the First Affiliated Hospital of Harbin Medical University. Written informed consent was obtained from all of the subjects before they were enrolled. All activities involving human subjects were conducted in accordance with the Declaration of Helsinki.

### 2.2. Tag SNPs of the MIR155HG/miR-155 Gene

**Figure 1:**
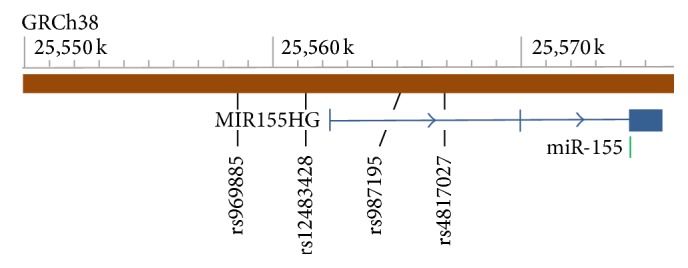
Loci of the MIR155HG/miR-155 gene and its tag SNPs. The three blue vertical lines/box and the two blue lines with arrows represent three exons and two introns of the MIR155HG gene, respectively. The miR-155 gene is encoded in the third exon of its host gene (MIR155HG). Regarding the 4 tag SNPs, rs969885 and rs12483428 are located in the predicted promoter region of the MIR155HG gene, whereas rs987195 and rs4817027 are located in the first intron of the MIR155HG gene.

Using the Chinese Han in Beijing (CHB) population of the International HapMap Project, we attempted to deduce tag SNPs of the MIR155HG/miR-155 gene, a group of particular SNPs that might represent all of the SNPs identified in the MIR155HG/miR-155 gene. Using Haploview 4.2 (with the following parameters: *r* ^2^ threshold: 0.8; HW *p* value cutoff: 0.05; minimum genotype: 95%; and minimum minor allele freq.: 0.05), 4 tag SNPs (rs969885, rs12483428, rs987195, and rs4817027) were selected, and their association with epilepsy was investigated. The loci of the MIR155HG/miR-155 gene and its tag SNPs are shown in Figure 1.

### 2.3. Subject Enrollment

A total of 249 epileptic patients (male/female: 137/112; mean age: 26.51 ± 15.25 years) and 289 healthy individuals (male/female: 157/132; mean age: 27.32 ± 21.24 years) were enrolled between 2011 and 2013. All of the subjects were Han Chinese. Of the total number of subjects, 134 (out of 249) epileptic patients and 177 (out of 289) healthy individuals were recruited from the Department of Neurology and the Health Management Center in the Affiliated Hospital of Guangdong Medical College in southern China. The remaining subjects were recruited from the Department of Neurology and the Health Management Center in the First Affiliated Hospital of Harbin Medical University in northern China. All of the epileptic patients were diagnosed according to the definition of epilepsy proposed by the International League Against Epilepsy (ILAE) [18] and then stratified according to gender (male/female), age of onset (early-onset epilepsy: <18 years; late-onset epilepsy: ≥18 years), temporal lobe epilepsy (TLE), and drug-resistant epilepsy (DRE). The inclusion criteria for TLE were mainly based on typical temporal auras and temporal discharges at onset using video-electroencephalograph (V-EEG), and 174 patients were selected into the TLE subgroup. According to the definition proposed by the ILAE in 2010 [19], DRE was defined as the absence of a change or a reduction in seizure frequency of <60% after at least one year of treatment with two or more tolerated, appropriately selected and canonically used AEDs schedule, and 67 cases were selected into the DRE subgroup. Additionally, 3 epileptic patients and 8 healthy volunteers whose blood samples failed to be detected for tag SNPs and/or CNVs were excluded from the study, as well as 5, 2, and 4 cases suffered from extrinsic factors including intracranial infections, tumors, and brain trauma, respectively.

### 2.4. DNA Extraction and Genotyping of the Tag SNPs

Peripheral blood samples were collected from all of the subjects. DNA was then extracted using blood Genomic DNA Extraction Kits (Tiangen Biotech, Beijing, China) and stored at −80°C before being used for genotyping. All the samples were genotyped for 4 tag SNPs (rs969885, rs12483428, rs987195, and rs4817027) of the MIR155HG/miR-155 gene using the ABI PRISM SNapShot method (Applied Biosystems, Carlsbad, USA). The forward and reverse primers used in multiple PCR for these SNPs were as follows: rs969885, 5′-GGTGGCAGGGACTGAACCATT-3′ (forward primer) and 5′-AGCATTGCATTTCCTTAAGAGTCTGAG-3′ (reverse primer); rs12483428, 5′-ATATGTCCTGGAGATGGGAGTG-3′ (forward primer) and 5′-ATCCCTACCTCATCACCCTTCA-3′ (reverse primer); rs987195, 5′-CCATCAGCCCTGGAGACACATC-3′ (forward primer) and 5′-GGAGGAACCAGTCCTGCTGACA-3′ (reverse primer); rs4817027, 5′-TCACCAAGCATTGATGACTGATGTC-3′ (forward primer) and 5′-GGAGTGTTCATTGTTCTGTCGTTTTCA-3′ (reverse primer). The primers used in multiple SNapShot PCR for these SNPs were as follows: rs969885, 5′-TTTTTTTTTTTTTTTTAGTGGTACTTACTTTGACTTACTTTGGACATA-3′; rs12483428, 5′-TTTTTTTTTTTTTTTTTTTTTTTTTTTTTTGGAGAATGTTGTTGAGGTCAAAA-3′; rs987195, 5′-TTTTTTTTTTTTGGTACTCTTGTCACAGCAGCCCTC-3′; and rs4817027, 5′-TGACAATGCAAAACATTTAGTTGGGTG-3′. In addition, 5% of the samples were randomly selected for quality control.

### 2.5. Copy Number Detection

The copy numbers of the MIR155HG/miR-155 gene were determined using Multiplex AccuCopy Kits (Genesky Biotechnologies Inc., Shanghai, China), and 4 reference genes (POP1, RPP14, POLR2A, and TBX15) were used for normalization. The basic principle is shown at http://biotech.geneskies.com/en/technology/service/205.html. The target fragment of the MIR155HG/miR-155 gene is 154 base pairs in length and maps to the miR-155 gene, and the forward and reverse primer binding regions (PBRs) were as follows: forward PBR, 5′-TAGGGGTTTTTGCCTCCAACTG-3′ and reverse PBR, 5′-CAAAGAAGCATGAGTCACCCTGC-3′. In addition, the competitive DNA fragment was synthesized and was nearly the same as the target fragment, except for the insertion of 2 extra base pairs. According to the AccuCopy Kit manual, 20 *μ*L of the PCR reaction mix was prepared for each sample; the mixture was composed of AccuCopy PCR Master Mix, Fluorescence Primer Mix, sample DNA, competitive DNA fragments of the target fragment and 4 reference genes, and forward/reverse primers. The PCR process was as follows: 95°C/10 min; 11 cycles of 94°C/20 s, 65°C/40 s, and 72°C/90 s; 24 cycles of 94°C/20 s, 59°C/30 s, and 72°C/90 s; and 60°C/60 min. Then, the products were analyzed using an ABI3730XL sequencer, and a sample/competitive (S/C) peak ratio was computed for the target fragment and the 4 reference genes. The S/C ratio for the target fragment was first standardized to the 4 reference genes. For each reference gene, the 4 standardized S/C ratios were further standardized to the median value of all of the samples and then averaged. If one of the 4 standardized S/C ratios deviated from the mean of the other 3 by more than 25%, the ratio was excluded from the study. Finally, the copy numbers of the target fragment were computed based on the mean S/C ratios × 2, which were considered the copy numbers of the MIR155HG/miR-155 gene.

### 2.6. Statistical Analyses

Age is displayed as the mean ± standard deviation (SD) and was compared using Student's *t*-test. The gender, allele, and genotype frequencies were compared between cases and controls using the Chi-squared test or Fisher's exact test, and the analyses were repeated after stratification based on gender, age of onset, TLE, and DRE. The statistical analyses were performed using SPSS 19.0 (IBM, New York, USA), and a two-tailed *p* ≤ 0.05 was considered statistically significant. Power analyses were performed using Quanto 1.2 (University of Southern California, Los Angeles, USA). In addition, haplotypes were deduced and compared using Haploview 4.2 (Daly Lab, Cambridge, USA).

## 3. Results

### 3.1. Subject Information

This study enrolled a total of 249 epileptic patients and 289 healthy individuals, and the subject information is shown in Table 1. No significant differences in gender or age were observed between the epileptic cases and the healthy controls (*p* = 0.872 and 0.325, resp.).

### 3.2. Tag SNPs of the MIR155HG/miR-155 Gene

The frequency distributions of each tag SNP (rs969885, rs12483428, rs987195, and rs4817027) of the MIR155HG/miR-155 gene in the case group and the control group complied with Hardy-Weinberg equilibrium (all *p* > 0.05, data not shown). Power analyses using a log-additive mode indicated that this study would have 86.83% power for rs969885, 85.17% power for rs12483428, 98.54% power for rs987195, and 96.55% power for rs4817027 to detect a genotype with an odds ratio of 1.7 at a significance level of 0.05. However, in this study, no significant differences in the alleles, genotypes, and haplotypes were observed between the cases and the controls for the 4 tag SNPs (all *p* > 0.05, data not shown).

### 3.3. Tag SNPs of the MIR155HG/miR-155 Gene after Gender Stratification

The frequency of the AA genotype at rs4817027 was significantly higher in the male cases than in the male controls (*p* = 0.035), indicating an increased risk of epilepsy in the male population (Table 2). However, no significant differences in the alleles and genotypes were observed for the other 3 tag SNPs between the male cases and the male controls. In addition, no significant differences in the alleles and genotypes were observed between the female cases and the female controls for the 4 tag SNPs (all *p* > 0.05, data not shown).

This study further explored the haplotypes of the 4 tag SNPs between the male cases and the male controls and found a haplotype block composed of 3 tag SNPs (rs12483428, rs987195, and rs4817027). The frequency of the TCA haplotype was higher in the male cases than in the male controls (28.1% versus 20.7%, *p* = 0.036), indicating an increased risk of epilepsy in the male population (Table 3). However, no significant differences in haplotypes were observed between the female cases and the female controls (all *p* > 0.05, data not shown).

### 3.4. Tag SNPs of the MIR155HG/miR-155 Gene after Age of Onset Stratification

No significant differences in the alleles and genotypes were observed between the early-onset/late-onset cases and the controls for the 4 tag SNPs (all *p* > 0.05, data not shown). This study further explored the haplotypes of the 4 tag SNPs between the early-onset cases and the controls and found a haplotype block composed of 2 tag SNPs (rs969885 and rs987195). The frequency of the CC haplotype was higher in the early-onset cases than in the controls (52.8% versus 43.6%, *p* = 0.011), indicating that the CC haplotype should be a genetic susceptibility factor for early-onset epilepsy (Table 3). However, no significant differences in haplotypes were observed between the late-onset cases and the controls (all *p* > 0.05, data not shown).

### 3.5. Tag SNPs of the MIR155HG/miR-155 Gene after TLE Stratification

No significant differences in the alleles and genotypes were observed between the TLE cases and the controls for the 4 tag SNPs (all *p* > 0.05, data not shown). This study further explored the haplotypes of the 4 tag SNPs and found a haplotype block composed of 2 tag SNPs (rs969885 and rs987195). However, no significant differences in haplotypes were observed between the TLE cases and the controls (all *p* > 0.05, data not shown).

### 3.6. Tag SNPs of the MIR155HG/miR-155 Gene after DRE Stratification

The frequency of the AA genotype at rs4817027 was significantly higher in the DRE cases than in the controls (*p* = 0.024), indicating that the AA genotype at rs4817027 is a genetic susceptibility factor for DRE (Table 4). However, no significant differences in the alleles and genotypes were observed for the other 3 tag SNPs.

This study further explored the haplotypes of the 4 tag SNPs between the DRE cases and the controls and found a haplotype block composed of 2 tag SNPs (rs969885 and rs987195). The frequency of the CC haplotype was higher in the DRE cases compared with the controls (59.0% versus 44.6%, *p* = 0.003), indicating an increased risk of DRE, whereas the frequency of the CG haplotype was lower in the DRE cases compared with the controls (29.9% versus 43.3%, *p* = 0.004), suggesting a protective effect against DRE (Table 3).

### 3.7. CNVs of the MIR155HG/miR-155 Gene

**Figure 2:**
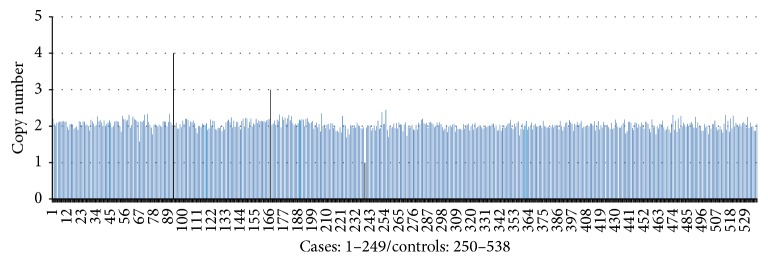
Copy numbers of the MIR155HG/miR-155 gene in a total of 538 individuals. Cases 93, 167, and 239, which exhibited CNVs, are shown by the 3 black vertical lines, and the others without CNVs are denoted by blue vertical lines.

A total of 3 epileptic cases with CNVs of the MIR155HG/miR-155 gene were observed in the case group, including 1 case with deletion (copy number: 1; 0.40%) and 2 cases with duplication (copy numbers: 3 and 4; 0.80%), but no CNVs were observed in the control group (Figure 2).

## 4. Discussion

In recent years, a large number of experimental and human studies of epilepsy have performed global expression profiling of miRNAs, and more than one hundred miRNAs have been found to be abnormally expressed in epileptic brains [20]. However, only a few miRNAs have been independently validated in more than one study, including miR-21, miR-34a, miR-132, miR-134, miR-146a, and miR-155. Functional studies using mimics or inhibitors have revealed their roles in the control of cell survival (miR-21, miR-34a, and miR-132), synaptic structure (miR-134), and inflammatory responses (miR-146a and miR-155) [21]. Obviously, miR-146a and miR-155 are the miRNAs that are most involved in the inflammatory process of epilepsy. More recently, a positive association between epilepsy and the miR-146a rs2910464 has been demonstrated in the Chinese population by our team [22]. However, the genetic association between epilepsy and miR-155, another inflammatory miRNA, remained unknown until this study, which provides the first report that MIR155HG/miR-155 tag SNPs are related to an increased risk of early-onset epilepsy, DRE, and seizures in males. Additionally, rare CNVs were exclusively found in epileptic patients.

Indeed, a systematic review of forty clinical reports verified the higher incidence of epilepsy in males compared with females [23]; this difference is often attributed to the higher prevalence of lesional epilepsy in males [24, 25]. Interestingly, increased vulnerability to the development of epilepsy was confirmed in male rats in an animal model of MTLE induced by lesional insult (hyperthermia) [26]. In contrast, the incidence of epilepsy induced by nonlesional insult (stress) was higher in female models [27], which is in line with two clinical studies of cryptogenic TLE characterized by a lack of obvious lesions [23, 25]. Moreover, the timescale of cerebral development is longer in boys; immature male brains are more likely to suffer from the potential insults of epileptogenesis in childhood [28], which is also consistent with the higher vulnerability of males to seizures. In this study, gender stratification revealed that the TCA haplotype (rs12483428-rs987195-rs4817027) and the AA genotype at rs4817027 were genetic susceptibility markers for epilepsy in the male patients. Given the inflammatory role of miR-155, these genetic findings indicate that seizures in male patients could be related to the miR-155-mediated modulation of the inflammatory insults of lesional epilepsy.

Early-onset epilepsy, an age-dependent epileptic syndrome, primarily results from genetic defects and inborn errors. Many of the types of early-onset epilepsy display overlapping clinical features, indicating that they share pathologies, such as metabolic and structural brain abnormalities [29, 30]. Recently, inflammation has been identified as a crucial mechanism in epilepsy, though little is known about its association with early-onset epilepsy. In particular, the expression of miR-155, an inflammatory modulator, is significantly increased in the brain in an immature rat model of status epilepticus and in children with MTLE [12], suggesting that the inflammatory role of miR-155 is involved in the development of early-onset epilepsy. This study further demonstrated that the CC haplotype (rs987195-rs969885) is a genetic susceptibility marker for early-onset epilepsy, confirming the role of miR-155 in the generation of early-onset epilepsy from a molecular genetics perspective.

Generally, the mechanisms of DRE involve the transporter hypothesis, the target hypothesis, and hypotheses involving drugs that fail to affect the real targets [31]; however, these mechanisms seem to be insufficient to explain clinical DRE. Recent studies have indicated that inflammation probably plays a crucial role in the development of DRE [32–35]. Moreover, inflammatory modulation could improve seizure control in some patients with DRE [34, 35], which supports the hypothesis of an inflammatory mechanism of DRE and suggests a new strategy for drug resistance. Interestingly, this study found that the AA genotype at rs4817027 and the CC haplotype (rs987195-rs969885) were genetic susceptibility markers for DRE but the CG haplotype (rs987195-rs969885) was a genetic protective factor against DRE. Based on the inflammatory role of miR-155 in the central nervous system, these genetic findings are compatible with the inflammatory mechanism of DRE.

Normally, two copies of human genes are present in autosomal regions. Duplication/deletion of DNA fragments can lead to significant increase/decrease in gene expression levels due to dosage effects of their copy numbers, which plays a key role in the generation of some genetic diseases [36, 37]. This study found 2 cases with duplication of the MIR155HG/miR-155 gene, but the incidence of duplication was relatively low in the case group (0.80%); thus we were forced to abandon statistical assessment of their association with epilepsy. Still, we can put forth a rough hypothesis that the expression of miR-155 may be upregulated in cases with duplication, resulting in seizures through promotion of the inflammatory response.

In addition, 1 case with deletion of the MIR155HG/miR-155 gene was observed. This observation was inconsistent with the increased expression of miR-155 in epileptic patients, suggesting that miR-155 may be involved in other biological processes. The gene encoding methyl CpG binding protein 2 (Mecp2) is another predicted target of miR-155, and any gain or loss in the expression of this gene over a narrow threshold level leads to neurological impairment [38, 39], such as in MECP2 duplication syndrome and Rett syndrome. These patients show a marked increase in susceptibility to epileptic seizures. Thus, another potential mechanism can be proposed: the MIR155HG/miR-155 CNVs likely play a role in the generation of epilepsy by indirectly altering the expression of Mecp2.

Several limitations of this study should be acknowledged. First, relatively few subjects were enrolled in this study, which possibly give rise to nonspecific results. However, the epileptic patients enrolled in this study were relatively young (mean age: 26.51 ± 15.25 years); thus almost all of them did not suffer from other related comorbidities, which limits the risk of nonspecific results. Second, although rs12483428 and rs969885 are located in the predicted promoter region of the MIR155HG/miR-155 gene, this study did not involve additional experiments to assess the expression of miR-155 and its predicted targets, such as SOCS1 and Mecp2, or their association with the potential functional haplotypes, including the TCA haplotype (rs12483428-rs987195-rs4817027) and the CC/CG haplotype (rs969885-rs987195) because obtaining tissue samples of epileptogenic foci is difficult. Moreover, the brain is a specialized tissue with its own resident immune system. The brain also differs from peripheral tissues due to the presence of the blood-brain barrier. Thus, this study did not include functional experiments based on peripheral blood samples. Third, all of the subjects in the study were Han Chinese, and caution should be used when generalizing these findings to different ethnic populations.

## 5. Conclusions

This study is the first to demonstrate that MIR155HG/miR-155 tag SNPs are associated with epilepsy. It also showed that rare CNVs were found exclusively in epileptic patients. These findings clarify the role of miR-155 in epilepsy from the perspective of molecular genetics.

## Figures and Tables

**Table 1:** Subject information.

	Cases	Controls	*p* value
Gender (male/female, *n*)	137/112	157/132	0.872
Age (mean ± SD, years)	26.51 ± 15.25	27.32 ± 21.24	0.325
Age of onset (*n*)			
Early-onset epilepsy	145	—	—
Late-onset epilepsy	104	—	—
TLE (*n*)	174	—	—
DRE (*n*)	67	—	—

**Table 2:** Frequency distributions of the 4 tag SNPs between the male cases and the male controls.

	Male cases *n* (%)	Male controls *n* (%)	OR (95% CI)	*p* value
rs969885 C>T				
C/T	238 (86.86)/36 (13.14)	266 (84.71)/48 (15.29)	0.80 (0.44–1.45)	0.463
CC/CT/TT	103 (75.18)/32 (23.36)/2 (1.46)	111 (70.70)/44 (28.03)/2 (1.27)		0.457
CC/CT+TT	103 (75.18)/34 (24.82)	111 (70.70)/46 (29.30)	0.82 (0.42–1.60)	0.564
CC+CT/TT	135 (98.54)/2 (1.46)	155 (98.73)/2 (1.27)	2.40 (0.29–19.94)	0.417
rs12483428 T>C				
T/C	241 (87.96)/33 (12.04)	270 (85.99)/44 (14.01)	1.40 (0.76–2.58)	0.276
TT/TC/CC	107 (78.10)/27 (19.71)/3 (2.19)	115 (73.25)/40 (25.48)/2 (1.27)		0.273
TT/TC+CC	107 (78.10)/30 (21.90)	115 (73.25)/42 (26.75)	1.51 (0.77–2.98)	0.232
TT+TC/CC	134 (97.81)/3 (2.19)	155 (98.73)/2 (1.27)	0.95 (0.09–9.60)	0.962
rs987195 C>G				
C/G	167 (60.95)/107 (33.05)	184 (58.60)/130 (41.40)	1.04 (0.68–1.59)	0.853
CC/CG/GG	51 (37.23)/65 (47.45)/21 (15.33)	55 (35.03)/74 (47.13)/28 (17.83)		0.858
CC/CG+GG	51 (37.23)/86 (62.77)	55 (35.03)/102 (64.97)	0.86 (0.47–1.57)	0.619
CC+CG/GG	116 (84.67)/21 (15.33)	129 (82.17)/28 (17.83)	1.46 (0.68–3.13)	0.335
rs4817027 G>A				
G/A	197 (71.90)/77 (28.10)	249 (79.30)/65 (20.70)	1.30 (0.80–2.11)	0.287
GG/GA/AA	70 (51.09)/57 (41.61)/10 (7.30)	94 (59.87)/61 (38.85)/2 (1.27)		0.252
GG/GA+AA	70 (51.09)/67 (48.91)	94 (59.87)/63 (40.13)	1.18 (0.66–2.13)	0.575
GG+GA/AA	127 (92.70)/10 (7.30)	155 (98.73)/2 (1.27)	9.40 (1.17–75.31)	0.035

OR: odds ratio; 95% CI: 95% confidence interval. OR (95% CI) and *p* values have been adjusted for age in the logistic regression analyses.

**Table 3:** Frequency distributions of the differential haplotype blocks of the MIR155HG/miR-155 gene.

	Haplotype	Frequency (%)	Case ratio (%)	Control ratio (%)	*p* value
Male cases versus male controls					
rs12483428-rs987195-rs4817027	TGG	40.31	39.05	41.40	0.562
	TCA	24.15	28.10	20.70	0.036
	TCG	22.45	20.80	23.89	0.372
	CCG	13.10	12.04	14.01	0.480
Early-onset cases versus controls					
rs969885-rs987195	CC	46.67	52.79	43.60	0.011
	CG	39.62	35.48	41.70	0.079
	TC	13.58	11.34	14.71	0.177
DRE cases versus controls					
rs969885-rs987195	CC	47.33	58.96	44.64	0.003
	CG	40.73	29.85	43.25	0.004
	TC	11.94	11.19	12.11	0.768

**Table 4:** Frequency distributions of the 4 tag SNPs between the DRE cases and the controls.

	DRE cases *n* (%)	Controls *n* (%)	OR (95% CI)	*p* value
rs969885 C>T				
C/T	119 (88.81)/15 (11.19)	499 (86.33)/79 (13.67)	0.79 (0.36–1.72)	0.548
CC/CT/TT	53 (79.10)/13 (19.40)/1 (1.49)	214 (74.05)/71 (24.57)/4 (1.38)		0.536
CC/CT+TT	53 (79.10)/14 (20.90)	214 (74.05)/75 (25.95)	0.85 (0.36–2.04)	0.717
CC+CT/TT	66 (98.51)/1 (1.49)	285 (98.62)/4 (1.38)	5.07 (0.35–73.13)	0.233
rs12483428 T>C				
T/C	106 (79.10)/28 (20.90)	496 (85.81)/82 (14.19)	1.27 (0.63–2.57)	0.511
TT/TC/CC	44 (65.67)/18 (26.87)/5 (7.46)	213 (73.70)/70 (24.22)/6 (2.08)		0.516
TT/TC+CC	44 (65.67)/23 (34.33)	213 (73.70)/76 (26.30)	1.29 (0.57–2.90)	0.542
TT+TC/CC	62 (92.54)/5 (7.46)	283 (97.92)/6 (2.08)	1.55 (0.16–14.83)	0.705
rs987195 C>G				
C/G	94 (70.15)/40 (29.85)	332 (57.44)/246 (42.56)	1.59 (0.92–2.75)	0.097
CC/CG/GG	34 (50.75)/26 (38.81)/7 (10.45)	100 (34.60)/132 (45.67)/57 (19.72)		0.118
CC/CG+GG	34 (50.75)/33 (49.25)	100 (34.60)/189 (65.40)	1.98 (1.92–4.28)	0.081
CC+CG/GG	60 (89.55)/7 (10.45)	232 (80.28)/57 (19.72)	1.48 (0.54–4.03)	0.448
rs4817027 G>A				
G/A	96 (71.64)/38 (28.36)	455 (78.72)/123 (21.28)	1.72 (0.91–3.24)	0.094
GG/GA/AA	34 (50.75)/28 (41.79)/5 (7.46)	174 (60.21)/107 (37.02)/8 (2.77)		0.074
GG/GA+AA	34 (50.75)/33 (49.25)	174 (60.21)/115 (39.79)	1.63 (0.76–3.52)	0.213
GG+GA/AA	62 (92.54)/5 (7.46)	281 (97.23)/8 (2.77)	13.13 (1.40–123.83)	0.024

OR: odds ratio; 95% CI: 95% confidence interval. OR (95% CI) and *p* values have been adjusted for gender and age in the logistic regression analyses.
